# 

**Published:** 1890-06

**Authors:** 


### CONTENTS—JUNE, 1890.—VOL. 5, NO. 12.

ORIGINAL COMMUNICATIONS—

Gun-Shot Wound of the Bladder—Laparotomy and Recovery. A. C. Walker, M. D. .......... 449

Mineral Waters of Texas. Edgar Everhart, Ph. D. ..... 453

CORRESPONDENCE—

Further Success with Potassii Nitras. L. W. Cock, M. D. . 463

Nitrate Potassa in Chills .......... 463

SOCIETY NOTES—

Terrell Medical Society—Abstract of Proceedings ........ 464

Cherokee Medical Society .......... 467

EDITORIAL—

Unpleasant, but a Duty, Nevertheless .......... 469

W. P. Burts, M. D., President Texas State Medical Association .......... 471

The End of Volume Five .......... 472

Special Notice .......... 473

Fiat Justitia .......... 474

MEDICAL NEWS AND MISCELLANY—

Containing Many Items of Interest .......... 474

BOOK NOTICES—

Notices and Reviews .......... 477

PUBLISHER'S NOTES—

Which Should be Read By All .......... 480

